# Atypical Brain MRI Findings in a Child With Delayed Diagnosis of Anti-N-Methyl-D-Aspartate Receptor Encephalitis

**DOI:** 10.7759/cureus.18103

**Published:** 2021-09-19

**Authors:** Saber Jan, Arayamparambil C Anilkumar

**Affiliations:** 1 Pediatrics, State University of New York Upstate Medical University, Syracuse, USA; 2 Neurology, State University of New York Upstate Medical University, Syracuse, USA

**Keywords:** biotin, thiamine, pediatric, acute encephalitis, encephalopathy, brain mri, bilateral thalamic disease, basal ganglia disease, anti-nmda, autoimmune encephalitis

## Abstract

Anti-N-methyl-D-aspartate receptor encephalitis (anti-NMDARE) is the most common cause of autoimmune encephalitis in children with a wide spectrum of clinical presentation and MRI findings. A high index of suspicion is required to avoid a delay in treatment and long-term morbidity. We present a healthy two-year-old male who developed fever and viral prodrome symptoms that rapidly progressed to acute encephalopathy, status epilepticus, quadriparesis, and abnormal movements. Brain MRI showed symmetric involvement of bilateral insula, posterior part of basal ganglia, and thalami. The patient survived the acute phase with supportive therapy but ended up with a devastating neurologic sequela, including developmental delay, inability to communicate, dysphagia, quadriparesis, and cortical visual impairment. Anti-N-methyl-D-aspartate (anti-NMDA) immunoglobulin G (IgG) antibodies were negative in serum and cerebrospinal fluid (CSF). The patient underwent an extensive inflammatory, infectious, metabolic, and genetic workup, including a whole-exome sequence (WES) and mitochondrial panel, which was unremarkable. CSF studies were unremarkable. Repeated anti-NMDA IgG antibodies were positive in serum a year after the presentation. This presentation highlights the crucial role of early immunotherapy in suspected autoimmune encephalitis (AE) cases, even at a young age, to prevent devastating neurologic outcomes. Moreover, clinicians should not rely on antibody results to treat a suspected case of AE due to possible false-negative test results, and the majority of AE cases remain without known antibodies.

## Introduction

Acute encephalitis in children has a broad clinical presentation and requires a high index of suspicion for early diagnosis and treatment. Anti-N-methyl-D-aspartate receptor encephalitis (anti-NMDARE) is well-characterized autoimmune encephalitis (AE) since it was first identified in 2007 by Dalmau and colleagues [1]. The presentation includes psychosis, memory loss, seizures, dyskinetic orofacial movement, ataxia, impaired level of consciousness, and autonomic instability. Although brain MRI is normal in up to 50% of AE cases, lesions at different brain areas have been reported, including the medial temporal lobe, cerebral cortex, cerebellum, thalamus, hippocampus, basal ganglia, brainstem, and rarely in the spinal cord [2,3]. We present a case of a two-year-old child who presented with acute encephalopathy with unusual brain MRI findings involving the cerebral cortex, bilateral insula, thalami, and basal ganglia, and unremarkable initial extensive workup including anti-N-methyl-D-aspartate (anti-NMDA) immunoglobulin G (IgG) antibodies, who was diagnosed with anti-NMDARE almost a year after his presentation.

## Case presentation

**Figure 1:**
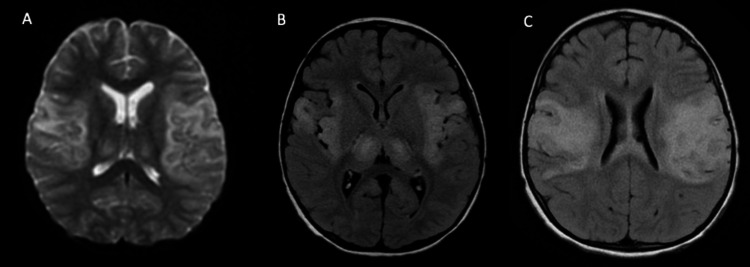
Brain MRI during the initial presentation. A: Axial DWI. B: Axial T2 FLAIR + GAD. C: Axial T2 FLAIR + GAD. DWI, diffusion-weighted imaging, FLAIR, fluid-attenuated inversion recovery, GAD, gadolinium.

A two-year-old Asian male, developmentally appropriate for age with a history of alpha thalassemia trait, presented to a tertiary hospital with fever and symptoms of viral prodrome. He then rapidly progressed to encephalopathy with an impaired level of consciousness, rhythmic arm movement, trismus, and salivary drooling. He was intubated, admitted to the pediatric intensive care unit (PICU) in July, and placed on video electroencephalogram (EEG) monitoring. Family history was not contributory and there was no consanguinity. A summary of extensive infectious, inflammatory, metabolic, and genetic workup performed is illustrated in Table 1. Cerebrospinal fluid (CSF) studies were unremarkable. Epstein-Barr virus (EBV) polymerase chain reaction (PCR) was positive in serum but negative in CSF. Moreover, EBV viral capsid antigen (VCA) immunoglobulin M (IgM) antibodies were negative too (Table 1). Anti-NMDA IgG antibodies were negative in serum and CSF. Video EEG showed diffuse slowing, absence of posterior dominant rhythm, and the movements identified as tonic seizure and epileptic spasms of uncertain onset. The patient was treated with anti-seizure medications, including levetiracetam and clobazam. Initial brain MRI showed diffusion restriction involving the left medial temporal lobe, bilateral insula, frontal and temporal opercular region, and bilateral thalami (Figure 1A). There was no blooming artifact to suggest hemorrhage. Magnetic resonance spectroscopy (MRS) was not performed. Magnetic resonance angiography and venography were normal. MRI of the cervical spine was normal. A follow-up brain MRI, in few days, showed symmetric signal abnormality over the bilateral insula and sub-insular regions, both lateral frontoparietal regions, posterior limb of the internal capsule, and anterior part of left mesial temporal lobe with extension to the body of corpus callosum (Figures 1B, 1C). After five days, the patient was medically stabilized and discharged from PICU to the pediatric floor then to a rehabilitation facility center. The patient had a devastating neurologic impairment, characterized by loss of age-appropriate developmental skills, inability to communicate, dysphagia, quadriparesis, poor head control, and cortical visual impairment (unable to fix or follow objects). He was able to breathe without assistance but was gastric tube dependent and unable to move without full assistance.

**Table 1:** Summary of primary investigations ^a^ Encephalitis panel included *Escherichia coli* k1, *Haemophilus influenzae*, *Listeria monocytogenes*, *Neisseria meningitidis*, *Streptococcus agalactiae*, *Streptococcus pneumoniae*, cytomegalovirus (CMV), enterovirus, herpes simplex virus 1, herpes simplex virus 2, human parechovirus, varicella-zoster virus, adenovirus DNA PCR, West Nile RNA PCR, EBV DNA PCR, human herpesvirus 6 DNA PCR, Eastern equine encephalitis virus (EEEV), and St. Louis encephalitis. PCR, polymerase chain reaction; IgM, immunoglobulin M; ELISA, enzyme-linked immunosorbent assay; Anti-NMDA, anti-N-methyl-D-aspartate; IgG, immunoglobulin G; GCDH, glutaryl-CoA dehydrogenase.

Test	Result	Reference range
Cerebrospinal fluid (CSF) studies		
White blood cells (WBC)	0	0-5/mcL
Red blood cells (RBC)	10	0-5/mcL
Protein	15 mg/dL	15-45 mg/dL
Glucose	75 mg/dL	60-80 mg/dL
CSF: Encephalitis panel PCR^a^	Negative	
Epstein-Barr virus (EBV) DNA PCR	Negative	
Mycoplasma pneumonia, PCR	Negative	
West Nile virus (WNV) IgM ELISA	Negative	
Oligoclonal bands	0	
Amino acid panel in CSF	Normal	
Anti-NMDA IgG in CSF	Negative	
Blood		
EBV quantitative PCR	111 IU/mL (high)	
EBV antibody viral capsid antigen (VCA), IgG	>600 U/ml (high)	0.0-17.9 U/mL
EBV antibody VCA, IgM	Negative	
EBV nuclear antigen antibody, IgG	>600 U/ml (high)	0.0-17.9 U/mL
*Toxoplasma gondii* IgG and IgM	Negative	
Human immunodeficiency virus (HIV) 1-2 combo antigen/antibody	Negative	
Mycoplasma pneumoniae IgM	Negative	
Lyme IgM/IgG	Negative	
Bartonella DNA PCR	Negative	
Erythrocyte sedimentation rate (ESR)	39 MM/HR (high)	0-10 MM/HR
C-reactive protein (CRP)	21	0.0-5.0 MG/L
Antinuclear antibody (ANA)	Negative	
Antineutrophil cytoplasmic antibodies (ANCA) panel	Negative	
Human leukocyte antigen (HLA)-B*51 allele	Negative	
Anti-NMDA IgG in serum (initial test)	Negative	
Anti-NMDA IgG in serum (after one year of presentation)	Positive 1:80	<1:10
Thyroid-stimulating hormone (TSH)	1.16 uIU/mL	0.400-4.200 uIU/mL
Fatty acid oxidation profile	Normal	
Homocysteine	Normal	
Acylcarnitine profile	Normal	
Amino acid panel	Unremarkable	
Lactate	Normal	
Ammonia	46 umol/L	9.00-30.00 umol/L
Next-generation sequencing of the GCDH gene associated with glutaric acidemia, type I	No variant detected	
Microarray	Normal Male XY,46	
Whole exome sequence (WES)	No pathogenic variant detected	
Mitochondrial genome panel	Negative	
Urine		
Urine organic acid	Unremarkable	
Drug screen	Negative	

As his family moved to our area, he was brought to our institution for establishing further care almost six months after the initial presentation. On neurological examination, he was awake with eyes open without any vocalization or response to commands. He had conjugate eye movements, but without any tracking or pursuit. He had spasticity with axial hypotonia. His hands remained fisted and he was moving right side more than left. He was in fixed flexion at the right knee and left leg in extension. He had hyperreflexia throughout.

**Figure 2:**
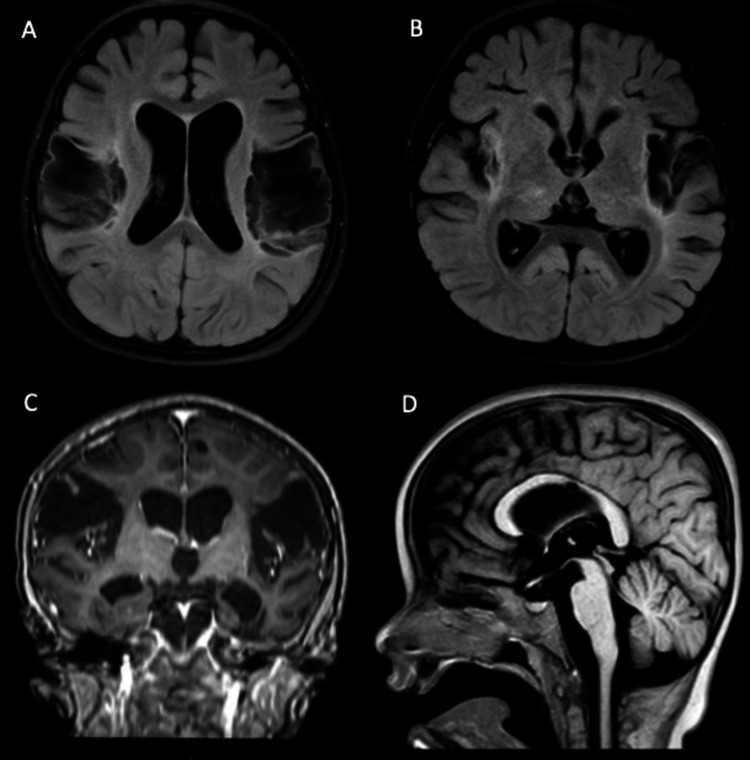
Follow-up brain MRI after one year of presentation. A: Axial T2 FLAIR. B: Axial T2 FLAIR. C: Coronal MPR. D: Sagittal T1 FLAIR. FLAIR, fluid-attenuated inversion recovery; MPR, multiplanar reformation.

At that point, he was wheelchair dependent, having spastic quadriparesis with continued spells of seizures despite the therapy with high doses of levetiracetam and clobazam. The patient was continued on clonidine, baclofen, and gabapentin. We started him on a trial of high dose biotin (5 mg/kg/day divided three times a day) and thiamine (100 mg three times a day) for suspected biotin-thiamine-responsive basal ganglia disease (BTBGD) despite the absence of pathogenic variants in the whole-exome sequence (WES) result given the devastating neurologic impairment and low side effect profile of biotin and thiamine. The caregiver reported a significant improvement in awareness, non-verbal communication, spontaneous movement, and spasticity. A mitochondrial genome panel tested was unremarkable. A follow-up brain MRI, almost a year after, did not show new brain lesions; however, interval development of cystic encephalomalacia was noted in the bilateral posterior frontal lobe, insular cortex, and bilateral thalami, and ventricles were noted to be more enlarged in size (Figures 2A, 2B). Moreover, volume loss was noted in the left hippocampus and body the of corpus callosum (Figures 2C, 2D). A repeated anti-NMDA IgG antibodies test in serum was positive a year after the initial presentation. Currently, the patient is three years old with stable neurologic impairment, seizure control, and unreported relapse since the initial presentation.

## Discussion

We describe a child with a delayed diagnosis of anti-NMDARE almost a year after the initial presentation of acute encephalitis associated with symptoms of viral illness, seizures, and devastating neurologic impairment. An extensive workup was unremarkable, including anti-NMDA IgG antibodies in serum and CSF. The case highlights the potential value of early empiric immunotherapy in a patient with suspected AE if the primary workup is unremarkable to avoid long-term morbidity.

Graus et al. published in 2016 a clinical approach to the diagnosis of AE, including anti-NDMARE, because of the broad spectrum of clinical presentation and the possibility of normal initial workup, including brain MRI and CSF studies [4]. The published criteria of AE diagnosis were refined in 2020 to address the difference between adult and pediatric presentation [5]. Probable antibody-negative pediatric AE remains a diagnostic challenge and requires careful exclusion of mimics without a delay of treatment [5,6]. It is well-known that the antibody of AE can remain unknown in up to 40-50% of cases [6,7]. Moreover, the false-negative result of anti-NMDA IgG antibodies, including CSF with commercial laboratory kit testing, has led to new laboratory techniques to improve the sensitivity of antibody testing [8,9].

Brain MRI in anti-NMDARE is normal in up to half AE cases. However, non-specific brain lesions at different brain regions and rarely in the spinal cord have been reported [2,3]. The highly symmetric involvement of bilateral insula and sub-insular regions, frontal and temporal opercular region, posterior part of basal ganglia, and thalami is an atypical finding in this case. A similar MRI finding has been reported in a pediatric patient following herpes simplex encephalitis who developed anti-NMDARE later without new brain lesions [10]. The involvement of bilateral thalami and basal ganglia has an extensive differential including toxic, metabolic (Leigh syndrome, glutaric aciduria type I, and others), infectious, inflammatory, vascular, and neoplastic causes [11]. Although there was no involvement of putamen or caudate in this case, biotin-thiamine-responsive basal ganglia disease (BTBGD), which is a treatable autosomal recessive condition affecting the solute carrier family 19 member 3 (SLC19A3) gene, should remain on differential [12]. Diffuse brain volume loss, including gray matter, basal ganglia, and hippocampus, has been reported in follow-up brain MRI in pediatric patients with anti-NMDARE [13]. This case showed left hippocampus and corpus callosum volume loss and enlarged ventricles in the follow-up brain MRI (Figures 2C, 2D).

In this case, the promising neurodevelopmental response to thiamine (vitamin B1) and biotin (vitamin B7) is encouraging for further clinical research for their role in autoimmune encephalitis. Although we acknowledge the limited evidence of a case report, thiamine has many different roles in the nervous system, such as anti-inflammatory, anti-oxidant, neuroprotective, neuro-conduction, and neuro-recovery effect [14,15]. Biotin has an important role in inflammatory and immune regulation, myelin regeneration, and reducing axonal hypoxia [16,17]. More clinical research is warranted to examine the effect of thiamine and biotin in treating neuroinflammatory conditions.

## Conclusions

Pediatric anti-NMDARE has a broad spectrum of clinical presentation and MRI findings. The differential of symmetric involvement of basal ganglia and thalami on brain MRI should include anti-NMDARE. Initial false-negative results of anti-NMDA IgG antibody, even in CSF, can be seen and should not delay immunotherapy if the patient meets the criteria of probable antibody-negative pediatric AE to avoid devastating neurologic sequelae.
